# A pathway to personal, population and planetary health for dietitians and nutrition professionals

**DOI:** 10.1002/puh2.137

**Published:** 2023-11-15

**Authors:** Kristen L. MacKenzie‐Shalders, Liza Barbour, Karen Charlton, Gregory R. Cox, Mark Lawrence, Sandra Murray, Kylie Newberry, Nicole M. Senior, Rosemary Stanton, Angela M. Tagtow

**Affiliations:** ^1^ Nutrition and Dietetics Research Group, Faculty of Health Sciences and Medicine Bond University University Drive Gold Coast Queensland Australia; ^2^ Department of Nutrition, Dietetics and Food, Faculty of Medicine Nursing and Health Sciences, Monash University Clayton Victoria Australia; ^3^ Indigenous and Health Sciences, Faculty of Science, Medicine and Health School of Medical, University of Wollongong Wollongong New South Wales Australia; ^4^ Institute for Physical Activity and Nutrition (IPAN) School of Exercise and Nutrition Sciences, Deakin University Geelong Victoria Australia; ^5^ School of Health Science, College of Health and Medicine, University of Tasmania Launceston Tasmania Australia; ^6^ Our Food System™ Brisbane Queensland Australia; ^7^ Professional Nutrition Services Sydney New South Wales Australia; ^8^ School of Medical Sciences, University of New South Wales Sydney New South Wales Australia; ^9^ Äkta Strategies, LLC University of Illinois Chicago, School of Public Health, Public Health Leadership Chicago Illinois USA

**Keywords:** climate, environment, food, health, indigenous peoples, planetary health, population health

## Abstract

**Background:**

Earth and all its inhabitants are threatened by a planetary crisis; including climate change, deforestation, biodiversity loss and pollution. Dietitians and nutrition professionals have a responsibility to lead transformational change in contemporary food and health systems to help mitigate this crisis. The study aims to develop a conceptual framework to support dietitians towards personal, population and planetary health.

**Methods:**

Non‐empirical methods were used by the co‐researchers to explore and explain the application of an international framework ‘Next‐Generation Solutions to Address Adaptive Challenges in Dietetics Practice: The I + PSE Conceptual Framework for Action’.

**Results:**

A non‐sequential pathway guide to personal, population and planetary health for nutrition professionals was developed including several key guiding principles of Agency, Action, Ascension, Alignment, Alliance and Allyship, and Advocacy and Activism. Each guiding principle features descriptors and descriptions to enhance dietitian and nutrition professional Agency (i.e. vision, self‐belief, confidence, strength and responsibility), Action (i.e. start, shift, translate, achieve and commit), Ascension (i.e. build, overcome, manage, challenge and progress), Alignment (i.e. leadership, transparency, diplomacy, values and systems), Alliance and Allyship (i.e. support, collaborate, represent, community and citizenship) and Advocacy and Activism (i.e. disrupt, co‐design, transform, empower and urgency). The framework and its descriptors support enhanced understanding and are modifiable and flexible in their application to guide the participation of dietitians and nutrition professionals in transformational change in personal, population and planetary health. This guide acknowledges that First Nations knowledge and customs are important to current and future work within this field.

**Conclusions:**

Alongside the international body of work progressing in this field, this framework and visual guide will support dietitians and nutrition professionals to achieve urgent, transformational change in personal, population and planetary health.

## INTRODUCTION

Our planet is in an ecological and climate crisis (herein ‘the planetary crisis’). Contemporary health and food systems – tasked to support individual health and nourishment – are forfeiting the health of future generations and the planet [1, 2, 3]. Urgent, transformative change from the status quo is needed [2, 3, 4, 5], including mitigation, addressing the root causes and adaptation to the current impacts of the planetary crisis [3, 4]. The world's population now surpasses 8 billion people, and all nutrition and health professionals have a responsibility to facilitate the essential changes needed in the world's health and food systems.

Dietitians and nutrition professionals have a critical role in promoting actions that supporting sustainable and resilient food and health systems [5, 6, 7]. The planetary crisis and other global changes, such as the COVID‐19 pandemic, have exposed systemic weaknesses, inequalities and adaptive challenges [2, 5, 8], and it is likely that nutrition and health professionals mirror broader societal anxiety, feelings of hopelessness, confusion, displaced blame and subsequent inaction [9, 10, 11, 12, 13, 14, 15, 16]. There are strong calls to action for dietitians and nutrition professionals to lead change and be ‘future‐focussed’ [17, 18, 19]. Dietitians and nutrition professionals work within complex health and food systems [2, 20–22], and the COVID‐19 pandemic has demonstrated that both these systems can transform at unprecedented rates [23, 24].

Both within Australia and the global context, there are examples of effective leadership to drive systemic changes in both the food and health systems. Examples of innovation and action within these sectors include strong commitments to net‐zero emissions by 2030–2040 [25, 26], toolkits for capacity building – including for nutrition and dietetic professionals – [27, 28] and policy and position statements [17, 29, 30]. By harnessing momentum for change, enabling structural change, growing public support and inspiring new supportive social norms, the Stockholm 2020 report suggests 2022 can be a new watershed moment for planetary health [16].

Planetary health (similar but non‐synonymous with one health and eco health) encompasses the disruptions of the Earth's natural systems, including climate change, deforestation, biodiversity loss and pollution, and their impact and association with public (human) health [1, 31–33]. Broadly, the term planetary health encompasses the connection of people, place, purpose and planet [8, 32]. First Nations peoples’ traditional knowledges are central to planetary health and healing [17, 34, 35]. Traditional First Nations or Indigenous territories encompass around 22% of the world's land surface and coincide with areas that hold 80% of the planet's biodiversity [36]. For example in Australia, the Environment Protection and Biodiversity Conservation Act 1999 (Section 3 [1]) recognises Aboriginal and Torres Strait Islander peoples’ knowledge and the need to work co‐operatively in the use of their knowledge to manage and protect the natural environment, including through ecologically sustainable development, the protection of biodiversity and the environment [37].

Dietitians and nutrition professionals have access to useful guides, toolkits and frameworks to support sustainable development more broadly [5, 32, 38], as well as an array of literature [5–7, 39–45]. However, the state of the planetary crisis requires a range of strategies and enhanced mobilisation of nutrition and dietetic resources to support planetary health; therefore, enhancing existing theory and synthesising divergent literature will be useful to support dietitians and nutrition professionals in their practice [46]. This article aims to develop a conceptual framework to support dietitians and nutrition professionals towards personal, population and planetary health, from a perspective of dietitians who are advocates for planetary health. The resulting conceptual framework will be designed to support enhanced understanding but be modifiable and flexible in its application [47] and guide the participation of dietitians and nutrition professionals in transformational change in personal, population and planetary health.

## METHODS

### Context

A non‐empirical approach was used to build upon existing theory [48] and develop a contextual framework. A conceptual framework is a network of interlinked concepts that together provide a comprehensive understanding of a phenomenon and support understanding [47]. The phenomenon of interest was supporting dietitians and nutrition professionals towards environmental sustainability and planetary health, grounded in practice and specific to a current context. The inductive approach was centred on the discussion of the theoretical framework ‘Next‐Generation Solutions to Address Adaptive Challenges in Dietetics Practice: The I + PSE Conceptual Framework for Action’ [5] due to its applicability to environmental sustainability and nutrition and dietetic practice and also involved dietitians perspectives and the synthesis of divergent literature into a new conceptual framework [46, 47].

As a non‐empirical perspective article from a co‐authorship team, no ethical clearance was required.

### Process

The lead investigator (KMS) identified and approached co‐authors who are advocates for planetary health and sustainable food systems in Australia, based on their teaching, research and communication activities, including an expert international collaborator from the United States of America. Each person was provided an opportunity to recommend other co‐authors, to an agreed limit (based on conventions of authorship) of 10 co‐authors to allow significant contribution. A First Nations dietitian was also engaged to consult on the conceptual framework and manuscript.

The process involved scholarly and theoretical discussion (February–April 2022), instigated by the lead author and between co‐authors, through video conferencing software and shared electronic documents. This involved consideration of ‘Next‐Generation Solutions to Address Adaptive Challenges in Dietetics Practice: The I + PSE Conceptual Framework for Action’ [5] dietitians perspectives and key, prioritised actions – and supporting resources – to guide dietitians and nutrition professionals towards environmental sustainability and planetary health in the current context.

The lead author (KMS) took written notes from the discussions and clarified them as required. From this process, inductive reasoning was used by extracting likely (but not certain) premises (concepts) from specific and limited observations. Similar theoretical concepts were grouped and interpreted, and given conceptual labels ‘i.e. themes’ [47] informed by Braun and Clarke's thematic method [49] and exemplar processes for building a conceptual framework [47, 50]. LB and KMS collaborated in familiarising and generating initial themes (hereinafter to be applicable to practice, these were referred to as guiding principles).

### Framework development

All co‐authors reviewed the guiding principles, and these were refined through iterative methods including repetitive synthesis and resynthesis [47] and named and re‐named by KMS in conjunction with NS and SM. Attributes, relationships, characteristics and definitions of guiding principles were discussed to inform descriptions [47, 51]. All co‐authors discussed and agreed on the final contextual framework title, the guiding principles and their descriptors. To ground in practice, examples and key resources specific to nutrition and dietetics were included. In addition, a visual summary (graphic) of the contextual framework was developed through consultation with an external illustrator featuring a sweet pea vine depicting nourishment and growth and illustrating the connectivity and inter‐directionality of the pathway guiding principles.

## RESULTS

**FIGURE 1:**
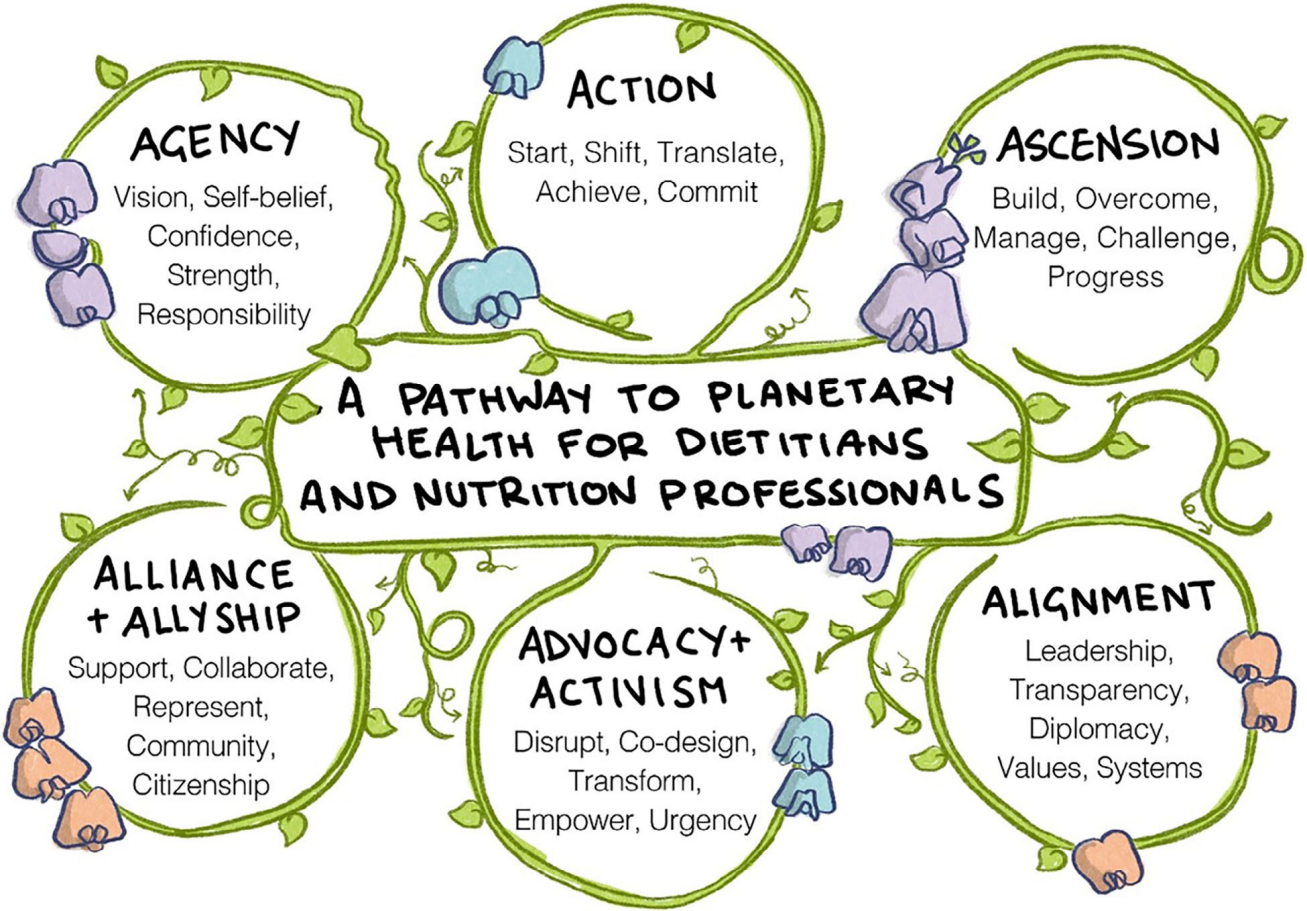
A pathway to personal, population and planetary health for dietitians and nutrition professionals featuring a sweet pea vine depicting nourishment and growth and illustrating the connectivity and inter‐directionality of the pathway guiding principles of Agency, Action, Ascension, Alignment, Alliance and Allyship, and Advocacy and Activism.

A non‐sequential pathway guide to personal, population and planetary health for nutrition professionals was developed, including several key guiding principles of Agency, Action, Ascension, Alignment, Alliance and Allyship and Advocacy and Activism. Each guiding principle features descriptors and descriptions to enhance dietitian and nutrition professional Agency (i.e. vision, self‐belief, confidence, strength and responsibility), Action (i.e. start, shift, translate, achieve and commit), Ascension (i.e. build, overcome, manage, challenge and progress), Alignment (i.e. leadership, transparency, diplomacy, values and systems), Alliance and Allyship (i.e. support, collaborate, represent, community and citizenship), and Advocacy and Activism (i.e. disrupt, co‐design, transform, empower and urgency). The non‐sequential principles and key descriptors are visually demonstrated in Figure 1.

The conceptual framework, key descriptors and description and dietetic practice examples are featured in Table 1.

**TABLE 1:** Pathway to planetary, population and planetary health guiding principles and descriptions.

Pathway guiding principles	Key descriptors	Description and dietetic practice examples
Agency	Vision Self‐belief Confidence Strength Responsibility	Vision to take our place in the collective movement Self‐belief and confidence as an individual, group or community Strengths‐based approaches to continual professional development, e.g. through existing resources and organisations Recognise own moral and ethical responsibility
Action	Start Shift Translate Achieve Commit	Start – however, you can Shift – yourself and others – to urgent action For Agenda 2030 (The Sustainable Development Goals) and other international commitments (e.g. towards net zero) Commit Translate Achieve e.g local, seasonal, plant‐forward approaches, address food waste in all forms and decreasing use of nutrient‐poor ultra‐processed foods
Ascension	Build Overcome Manage Challenge Progress	Build success. Overcome and manage anxiety, barriers and obstacles Challenge the status quo and wicked problems Progress, e.g. Smaller to bigger Personal to collective Local to global Simple to challenging Reactive to proactive
Alignment	Leadership Transparency Diplomacy Values Systems	Transparency of vision and values Practice diplomacy, authenticity and leadership Apply systems thinking, problem solving and approaches. Recognise interconnectivity of personal, population and planetary health Align Individual values, knowledge, skills and behaviours Personal and professional Policy, process and people
Alliance and Allyship	Support Collaborate Represent Community Citizenship	Engage in supportive, positive and safe community relationships Contribute to transformative, systemic change through collaboration, representation and collective problem solving Encourage, acknowledge and support First Nations peoples’ knowledge and leadership Be a global citizen
Advocacy and Activism	Disrupt Co‐design Transform Empower Urgency	Co‐design transformational planetary health solutions. Support Aboriginal and Torres Strait Islander peoples for planetary health projects and work they are prioritising Apply own privilege, position and capacity to act and empower others Advocate for equity and stand with and for disproportionately impacted communities Disrupt and act urgently including outside of own comfort level and norms, e.g. political action, protests, demonstration and lobbying

Key representative resources to support dietitians’ professional development in environmental sustainability and planetary health were identified by the co‐authorship team and are featured in Table 2. Please note that these resources are examples and are not designed to be complete or presented in a prioritised order.

**TABLE 2:** Key resources to support dietitians’ professional development in environmental sustainability and planetary health.

Resource	Organisation	Location	Synopsis
Food systems and environmental sustainability role statement [52]	Dietitians Australia	Australia	Lists the knowledge and skills required by an Accredited Practising Dietitian (APD) to progress environmental sustainability of food systems and planetary health
Sustainability toolkit [53]	International Confederation of Dietetic Associations (ICDA)	International	A toolkit and portal to existing resources to support nutrition and dietetic professionals to integrate sustainability into their practice. Some key features include a self‐assessment/professional development tool, resources, case studies, infographics, learning modules a community of practice, recent updates and monthly GROW newsletter
Healthy, regenerative and just: framework for a national strategy on climate, health and well‐being for Australia [54]	Climate and Health Alliance (CAHA)	Australia	A comprehensive roadmap to support the Australian Government in taking a leadership role in protecting the health and well‐being of Australian communities from climate change and also in fulfilling its international obligations, including the Paris Agreement
One Blue Dot [28]	British Dietetic Association	United Kingdom	A web‐based toolkit which includes information, graphics, tools and links to help dietitians understand environmentally sustainable diets and discuss these with patients and clients. Some key features include frequently asked questions, a glossary, educational resources and practical meal swaps
Sustainable Food Systems Primer for RDNS and NDTRs [55]	Academy of Nutrition and Dietetics	United States of America	A web‐based introductory resource in a modular format that includes foundational knowledge, vocabulary and critical thinking skills to equip nutrition and dietetics practitioners to understand sustainable food systems. It features 7 × ∼20 min modules (approx. 2 h) and handout linking to other resources
Academy of Nutrition and Dietetics: Revised 2020 Standards of Professional Performance for Registered Dietitian Nutritionists [27]	Academy of Nutrition and Dietetics	United States of America	Six standards of Professional Performance with specific indicators and measurable action statements that guide how dietitians can apply the principles of sustainable food systems to a variety of practice settings
Health and Climate Change Toolkit [56]	World Health Organisation	International	Includes resources, policy and information related to climate change for a broad range of health professions. Some features include health impacts and co‐benefits, building resilience, vulnerability and adaptation assessments, educational material and national adaptation strategies and plans
The 2030 Agenda for Sustainable Development (The Sustainable Development Goals) [57]	United Nations	International	An internationally recognised blueprint for sustainable development, which many countries and organisations are signatory to and includes implementation progress reports. They include 17 goals and 169 targets and additional events, publications and actions. Includes a ‘lazy persons’ guide to saving the world’ that guides individuals
Next‐Generation Solutions to Address Adaptive Challenges in Dietetics Practice: The I + PSE Conceptual Framework for Action [5]		United States of America	A framework to guide dietitians to better address complex challenges and to formulate multidimensional strategies that optimise individual, population and planetary health

## DISCUSSION

This article presents a conceptual framework guide designed to support dietitians and nutrition professionals towards transformational change in personal, population and planetary health. Dietitians and nutrition professionals are well‐positioned within food and health systems to create change. Dietitians and nutrition professionals work in diverse practice areas that span many sectors relevant to food systems. These include agriculture and food production, processing, marketing and retail settings; health care and private clinical practice settings; sports; community‐based settings; public health and policy settings; food service management settings; and research and academic settings [27].

Conceptually, some the elements of this framework are not new, but this framework summarises and synthesises key information of relevance to dietitians and nutrition professionals in their professional practice – to support planetary health and to address the ecological crisis in the current context. The time has never been better for change [16], and – similar to the range of behaviour change frameworks which may be familiar [58, 59, 60] – this framework is designed to move dietitians and nutrition professionals from contemplation to action. It is also designed to support dietitians and nutrition professionals to recognise their capability and role in leading and supporting positive change. This conceptual framework contains similar elements to the health promotion strategies of the Ottawa Charter, namely to advocate (to boost the factors which encourage health), enable (allowing all people to achieve health equity) and mediate (through collaboration across all sectors) to promote personal, population and planetary health [61].

To our knowledge, the concept of agency has not commonly been used or applied in nutrition and dietetic practice. However, agency – or positive self‐belief – is an important starting point for positive change. There are barriers to progress, including political, economic or human factors outweighing planetary concerns, displaced blame, inequities, insecurities and climate‐anxiety and despair [9, 11–14], but we recognise action is a key antidote to despair. With environmental concerns, there is a well‐known attitude‐behaviour gap between some people's environmental beliefs and their actions [10, 62]. In such cases, this conceptual framework can support dietitians and nutrition professionals to model positive behaviours and promote positive action.

A key component of this framework is for dietitians and nutrition professionals to apply their current skillset and knowledge. As an example, dietitians and nutrition professionals can support positive change by translating dietary guidelines through a planetary health lens as in many cases, with appropriate training can support recommendations that support an individual's health which also support the environment [2, 63]. Dietitians and nutrition professionals can also upskill by understanding the complexity of the food system and its involvement in sustainable agriculture, the environmental impact of our food and health systems as well as touchpoints for change, broader climate mitigation and adaptation strategies, supporting food literacy (including environment) and how to select more sustainable options among sources of animal protein.

We recognise that any discussion on planetary health cannot reasonably occur without acknowledging the role that First Nations People as the traditional owners, carers and custodians of the lands on which we live and work, the Australian Aboriginal and Torres Strait Islander Peoples. Alongside this, we are grateful for the wisdom and contribution of a First Nations dietitian consultant who has contributed knowledge and cultural experience. We recognise that this is a preliminary conceptual framework; and that further collaboration and future work relating to planetary health and sustainability with First Nations people is vital. We propose to advance this body of work by supporting and collaborating with First Nations people in the work they are doing to support our planetary health and ensuring sustainable food sources.

We acknowledge that planetary health is a complex topic. This article has not been developed with a full review of evidence; instead, we have taken a non‐empirical approach which can be beneficial to guide practice [64]. Through this process, collective knowledge, a key framework and scholarly evidence have been interpreted to guide dietitians and nutrition professionals in practice. The resulting conceptual framework and commentary do not include specific or prescriptive dietary recommendations and the authors recommend that dietitians and nutrition professionals refer to appropriate national food guides, or other international sources of information as highlighted in Table 2. The framework and its descriptors are designed to support enhanced understanding and are modifiable and flexible in their application to guide the participation of dietitians and nutrition professionals in transformational change in personal, population and planetary health.

We acknowledge that the developed pathway is non‐hierarchical and circular and does not incorporate elements of assessing, formulating and implementing solutions and evaluating impact. Conceptual frameworks are indeterminist in nature and do not enable us to predict an outcome [47]. However, in this rapidly evolving field and as a logical next step to the development of a theoretical framework, further proof‐of‐concept work to evaluate the validation and implementation of this framework including its feasibility, scalability and translatability will be useful [47, 50].

It is acknowledged that other frameworks – which are not specific to but are applicable to the phenomenon of interest – are further developed and have this complexity and scope; for example, The I + PSE Conceptual Framework that guided the current study incorporates systems thinking, reflection and evaluation with broader application [5]. It includes three phases; assess determinants of health (including planetary health), formulate and implement (multi‐dimensional) solutions and evaluate impacts [5]. In addition, the United Nations Sustainable Development Goals (as listed alongside other resources in Table 2) and its five Ps (People, Planet, Prosperity, Peace and Partnerships) are key to informing action and holding signatory governments to account to meet a range of measurable targets, many of which are useful for dietitians and nutrition professionals [57]. For example, the Sustainable Development Goal Number 12 (Responsible Consumption and Production) specifically recognises and incorporates targets for food waste as a critical priority.

Alongside the developed conceptual framework, other frameworks have also been developed for dietitians and nutrition professionals which may be useful. These include recommendations from Dietitians of Canada (2020) to be reflexive, get involved, disseminate and advocate, and communicate by building relationships and partnerships, acting with partners and disseminating and advocating for change. The Food Citizenship framework (herein Food Citizenship) can also be applied to support a democratic, socially, and economically just, and environmentally sustainable food system [38]. Food citizenship conceptually recognises that the distinction between consumer and citizen is important and necessary to shape future food systems. It identifies that individuals have the power to shift from passive consumers, in which their purchasing behaviours are largely influenced by personal decisions [65] through to food citizens, who actively participate in reorientating the food system, recognising that membership of society comes with rights and responsibilities [66, 67]. To support the transformation of the food system, dietitians and nutrition professionals are called to adopt a food citizen mindset and become more knowledgeable about food and the food system [68], including its ecological footprint.

## CONCLUSION

This non‐empirical article has synthesised guidance from dietitians who are advocates for planetary health. A resulting conceptual framework guide ‘A Pathway to Planetary Health for Dietitians and Nutrition Professionals’, and its guiding principles of Agency, Action, Ascension, Alignment, Alliance and Allyship, and Advocacy and Activism, has been designed to support dietitians and nutrition professionals towards transformational change in personal, population and planetary health. Alongside the international body of work progressing in this field, this pathway guide is designed to support dietitians and nutrition professionals to lead transformative, positive change within our food and health systems and within the broader community.

## AUTHOR CONTRIBUTIONS

The non‐empirical study design was conceived by Kristen L. MacKenzie‐Shalders in consultation with Liza Barbour, Karen Charlton, Gregory R. Cox, Mark Lawrence, Sandra Murray, Kylie Newberry, Nicole M. Senior, Rosemary Stanton and Angela M. Tagtow. Discussion was led by Kristen L. MacKenzie‐Shalders in consultation with the co‐authorship team, and data analysis and interpretation was completed by Kristen L. MacKenzie‐Shalders and Liza Barbour, with Nicole M. Senior and Sandra Murray. All co‐authors reviewed and refined the final framework. The manuscript was written by Kristen L. MacKenzie‐Shalders with support from all co‐authors. The First Nations Consultant reviewed and provided feedback on the draft and final framework and manuscript. All co‐authors reviewed the manuscript and approved the final submission. All authors declare that this work has not been previously published.

## CONFLICT OF INTEREST STATEMENT

Nicole M Senior provides freelance consulting services to the food sector. All other authors declare no conflicts of interest.

## FUNDING INFORMATION

This research received no specific grant from any funding agency in the public, commercial or not‐for‐profit sectors. The First Nations Consultant received a consultancy stipend from the Bond University Master of Nutrition and Dietetic Practice Programme.

## Data Availability

Data sharing not applicable – the article describes entirely theoretical research.
